# Restructuring the built environment to change adult health behaviors: a scoping review integrated with behavior change frameworks

**DOI:** 10.1080/23748834.2019.1574954

**Published:** 2019-02-20

**Authors:** Stephanie Wilkie, Tim Townshend, Emine Thompson, Jonathan Ling

**Affiliations:** a School of Psychology, Faculty of Health Sciences and Wellbeing, University of Sunderland, Sunderland, UK; b School of Architecture, Planning and Landscape, Newcastle University, Newcastle, UK; c Department of Architecture and Built Environment, Northumbria University, Newcastle Upon Tyne, UK; d School of Nursing and Health Sciences, Faculty of Health Sciences and Wellbeing, University of Sunderland, Sunderland, UK

**Keywords:** Built environment, behavior change, COM-B, physical activity, public health, urban planning

## Abstract

Built environment restructuring can improve public health through increased opportunity for healthy behaviors. Behavioral science targets individual health behaviors within place, suggesting the potential to integrate these approaches. This scoping review was one of the first to summarise the impact built environment restructuring has on health outcomes and behaviors *and* integrate these findings with the Capability-Opportunity-Motivation-Behavior model and Theoretical Domains Framework of behavior change. Potential studies were identified from 12 academic databases in urban design, psychology and public health. Search parameters involved 50 environment types, for example green space or healthy cities, combined with both an intervention (e.g. green infrastructure, active transport) and a measurable health outcome (e.g. exercise, wellbeing). Searches were limited to North America, Europe, or Australia/New Zealand. Of 536 potential studies reviewed against defined inclusion/exclusion criteria, 23 contributed to the findings. Evidence supported the positive influence of restructuring on varied health outcomes, many of which were drivers and domains of health behavior. Most studies indicated a clear contribution to increased physical activity. Recommendations include the need for explicit communication of theories guiding restructuring project design, consideration of health outcomes beyond physical activity, and better investigation of unanticipated barriers to health behaviors arising from built environment restructuring projects.

## Introduction

The built environment refers to ‘homes, schools, workplaces, parks/recreation areas, business areas and roads…. all buildings and spaces and products that are created or modified by people’ (Srinivasan *et al*. 2003, p. 1446). These places can influence population health (Barton and Grant 2006, 2013, Policy Connect 2017, World Health Organization (WHO) 2016, 2017), for example with effects on diabetes (Müeller-Riemenschneider *et al*. 2013), respiratory disease (Song *et al*. 2017), heart disease (Yitshak-Sade *et al*. 2017) and obesity (Mackenbach *et al*. 2014). The built environment also has an indirect influence on public health by providing or constraining opportunities for physical activity (Sallis *et al*. 2016), through food environments that encourage or discourage healthy diets (Lake and Townshend 2006, Townshend and Lake 2009, Algert *et al*. 2016, Townshend 2017), or by facilitating relaxation and recreation (Irvine *et al*. 2013, Völker and Kistermann 2015).

One approach to improve health outcomes is to provide more opportunity for healthy behaviors through built environment restructuring and urban planning (Barton and Grant 2006, Chriqui *et al*. 2016). In addition, behavioral science can be used to target individual health behaviors within these built environments (Davis *et al*. 2015, Glanz and Bishop 2010, Michie *et al*. 2013, Quigley 2013). However, the extent to which urban planning and behavioral science evidence intersect is unclear. This is possibly due to cross-disciplinary differences in methodology and targeted level of influence (Barton and Grant 2006, 2013, Tate et al. 2016). Recently, several studies suggested potential for the integration of these approaches; but were limited because there was no specific focus on built environment restructuring (Hollands *et al*. 2013) or the focus was on only one type of restructuring intervention (Arnott *et al*. 2014, Roberts *et al*. 2016). Building on this work, we conducted a scoping review of varied built environment restructuring projects for their impact on health outcomes and behaviors that are commonly targeted in behavioral science.

Behavioral science and behavior change theories cross disciplines such as psychology, economics, and marketing, highlighting a multitude of factors that influence individual behavior (Glanz and Bishop 2010, Matjasko *et al*. 2016); and research has implemented over 80 theories (Davis *et al*. 2015). Health psychologists and practitioners developed the Theoretical Domains Framework (TDF) to consolidate 33 of the most frequently used of these theories to identify 14 behaviour change domains, each ‘encompassing a set of similar theoretical constructs’ (Cane *et al*. 2012, p. 2). An example of a domain is a social influence, which includes a variety of constructs such as social support, group norms, or feedback. One model with increasing application within behaviour change research in recent years is the Capability-Opportunity-Motivation-Behavior model (COM-B), which suggests behaviour is the result of these three processes (Michie *et al*. 2011, 2014). *Capability* refers to necessary physical and psychological resources, *opportunity* to influences beyond the individual that facilitate or hinder behavior, and *motivation* to the varied influences on decision-making. An abridged overview of the COM-B and TDF is provided in Table 1; readers are encouraged to refer to the original articles for a full account of each.

**Table 1.** Overview of the theory domain framework and capability-opportunity-motivation-behavior model of behavior change.

Theoretical Domains Framework^a^	Capability-Opportunity-Motivation Model^b^
TDF	COM-B
A framework of 14 domains (higher-order theoretical constructs) identified as being implemented across behavior change interventions in varied contexts including health.	Model consisting of three components for behavior change. The authors suggest capability and opportunity influence motivation. Behavior change is a bi-directional process by which all components influence the occurrence of the desired behavior; the occurence of the behavior can conversely contribute to perceptions of the components.
Knowledge (procedural, condition)	Capability (p.5)
	‘Actual capacity to engage in the behaviour.’
Skills (competence, development)	
Environmental context and resources (stressors, facilitators)	Opportunity (p. 5)
	All factors external to the individual that make the behavior possible or prompt it.’
Social influence (social pressure, support)	
Intention (intrinsic motivation, commitment)	Motivation (p. 5)
	“Brain processes that energize and direct
Behavioural regulation (habits, monitoring)	Behaviour.”

^a^Cane *et al*. (2012). A subset of the 14 domains are listed here and examples of related constructs identified by Cane *et al*. are provided in (). For a full account of all domains, their constituent constructs, and how each domain corresponds to the COM-B model, refer to the original publication (pp. 8-10).

^b^Michie *et al*. (2011). Definitions of each model component are the original authors. For a full account of each component, refer to the original publication.

The COM-B and TDF were chosen as frameworks for this review because both highlight the important role of the environmental context, resources, and restructuring in changing health behaviors (Michie *et al*. 2011, Cane *et al*. 2012). The COM-B model was developed as a response to perceived limitations with existing models (Michie *et al*. 2011). Specifically, it encompasses varying levels of behavioral influence ranging from individual through to broader cultural, environmental and societal factors incorporated into a broader behavior change wheel to improve the design of behavior change interventions. The COM-B sits at the center of the wheel, contextualised by intervention function and policy typologies. In this regard, it was well suited to built environment restructuring initiatives that also vary in function and policy context. It was also integrated with the TDF framework to illustrate how the COM-B links explicitly to each theoretical domain. Additionally, the intention of the TDF was to provide a structured approach that would facilitate cross-disciplinary use of behavior change concepts by researchers from other professions (Cane *et al*. 2012).

In health behavior research, behavioral influences are often broadly categorized as micro or macro-level (Swinburn *et al*. 1999, Backholer *et al*. 2014). Macro-level factors include services or infrastructures across sectors such as public transportation systems; micro-level factors range from those in an immediate, specific location (e.g. within the home, the local doctor’s office) to neighbourhood or citywide initiatives like the introduction of cycle paths that operate at a larger spatial scale to impact daily activity (Swinburn *et al*. 1999, Hollands *et al*. 2013). The investigation of the complex interplay between human health and the built environment is well established in human geography, urban design/planning, and environmental psychology; and the micro and macro-level influences used in health behaviour research have clear parity with socio-ecological frameworks in these disciplines (Barton and Grant 2006, 2013, Sallis *et al*. 2006). However, explicit integration of behavioral science approaches typically operating at the individual level with the built environment approaches operating at higher micro- and macro-levels of influence has been limited to date.

Consequently, the aim of this scoping review was to determine whether the two approaches could be integrated. To achieve this aim, we focused on studies reporting built environment restructuring projects, which are considered interventions in TDF (Cane *et al*. 2012) and a type of behavior change technique (Michie *et al*. 2013). All studies also reported measurable outcomes relevant to behavior change; therefore, successfully achieving this aim would be evidenced, where possible, the findings could be integrated with the COM-B and TDF.

Varied methods are used to survey existing literature; and the manner by which information is reported and the degree of quality assessment in each varies (Garritty *et al*. 2016). The most rigorous is the systematic review, which typically focuses on the effectiveness clinical interventions, requires between 6–24 months to complete, implements very specific quantitative methods often including meta-analysis, and involves critical quality assessment of the evidence (Kanguara *et al*. 2012). Other rapid evidence assessment methods aim to balance the need for scientific rigor with the often time-limited requirements of the users of this information (Tricco *et al*. 2015). There is no agreed definition for rapid evidence assessments (Abou-Setta *et al*. 2016, Tricco *et al*. 2015). Generally they occur over a short time frame (3–6 months) using streamlined steps based on those for systematic reviews, for example by only searching one or limited numbers of academic databases, having one instead of two researchers extract data (Tricco *et al*. 2016), and/or excluding study quality assessment (Arksey and O’Malley 2005, Peters *et al*. 2015). A specific type of rapid evidence assessment is the scoping review, ‘a form of knowledge synthesis that addresses an exploratory research question aimed at mapping key concepts, types of evidence, and gaps in research related to a defined area or field by systematically searching, selecting, and synthesizing existing knowledge’ (Colquhoun *et al*. 2014, p. 1294). The scoping review was chosen because it was consistent with the aim of mapping concepts and evidence across disparate disciplines in an exploratory manner.

## Methods

The review presented here was part of a wider literature review commissioned by the Public Health England Behavioural Insights Team to inform future research priorities. The methodology implemented five steps for scoping reviews: research question identification, identification of potential studies, inclusion/exclusion review, data charting, and findings/recommendations (e.g. Arksey and O’Malley 2005, Tricco *et al*. 2016). The funder specified the research question guiding the wider literature review: *To what extent have built environment restructuring projects affected adult health outcomes and behaviors commonly used in behavioral science?* Research inclusion/exclusion parameters and search terms were developed in conjunction with the funder. The authors independently generated the findings and recommendations.

To identify potential studies, the following databases were searched: Cochrane Library, Environmental Periodicals, PsycArticles, ProQuest, PubMed, SCOPUS, Social Sciences Index, SocINDEX, Thomson Reuters: Arts and Humanities Search, Urban Studies Abstracts, and Web of Science. During the search conducted between April–May 2017, three authors (A1, A2, A3) focused on the databases most relevant to their profession and implemented an iterative procedure with regular discussions to ensure consistent search methodology. The search included English-language studies with adult participants published between January 2000–March 2017. We chose 2000 as the starting point of our search because it allowed some time for the World Health Organization’s Healthy City Movement, initiated in the late 1980s/early 1990’s (Tsouros 1991), as well as the highlighted need for work linking built environments and public health at this time (e.g. Flynn 1996, Perdue *et al*. 2003) to be realised within both urban design and subsequent academic reporting. Searches were also limited to studies set in North American, Europe, or Australia/New Zealand as they have broadly similar urban design approaches to those in the UK, where the funder was based (Carmona *et al*. 2010).

Table 2 provides a summary of terms combined during the search process. This involved combining each lived environment search term with each intervention and each measurable outcome (e.g. urban AND active transport AND physical activity; urban AND active transport AND wellbeing). Built environment terms and project types included micro-level built projects *beyond the immediate, specific spatial scale* and macro-level projects (Swinburn *et al*. 1999, Backholer *et al*. 2014). Studies were excluded if they focused only on micro-level projects (e.g. within the home) or solely reported population-level trends associated with built environment characteristics. Measurable behaviours and outcomes were based on public health indicators such as obesity, physical activity and wellbeing (Department of Health 2015) and behavior change influences such as emotion, self-esteem, and social influence (Cane *et al*. 2012). Outcomes potentially related to *capability* and *motivation* and *social opportunity* aspects of the COM-B model were considered particularly important for searching based on the premise that built environment restructuring *is intended to provide physical opportunity* for health behaviors by default. Studies without measured behaviors or health outcomes were excluded (e.g. solely focused on subjective environment perceptions or social outcomes), as were studies conducted with child-only samples because adults often determine their experience in these settings.

**Table 2.** Scoping review database search terms.

Lived environments	Interventions	Measurable behaviours/outcomes linked to public health
Air quality Allotments Blue space* /City Cities, Towns Community gardens Country (nationality, not rural) Dene Dementia-friendly Districts /Eco- Eco-park Eco-town /Green Green field* Green space* Healthy cities /House Housing, Home, Housing plus Sheltered accommodation Inclusive design Lake* /Landscape Landscape planning Neighbour/neighbourhood New Urbanism Obesogenic Outdoor Park* Playing fields Promenade Public realm Region Responsive environments Resilient communities Salutogenic Seaside State Therapeutic landscape Town planning Townscape Transportation Urban Walkable /Water Waterfronts /Work Workplace, Worksite, Employment, Employer Occupational	Behavio* insight Behavio* economics ‘Behavio* change’ ‘Behavior change technique taxonomy (BCCTv1)’ /COM-B Capability Motivation Opportunity Nudge theory Nudge Ability Accessibility /Active transport Paths/Footpaths Cycle lanes Trails Availability Competence Crime prevention /Environment* Ambient temperature Characteristics Noise Pollution Stressors Floorscaping Green infrastructure Healthy towns /Influenc* ‘Social influen*’ ‘Social norms’ Knowledge Landscaping Lighting Mobility Psycholog* ‘Psychosocial factors’ Reinforcement Rewards Skill Street furniture Tree planting Vegetation	Alcohol Chronic conditions Diabetes Drug use Environmental attitude* Emotion Exercise Falls /Fatigue Tired* Injuries Health Health behaviour /Health-related quality of life Physical Mental /Medication Medication reduction /Mental health Anxiety, depression Mobility /Obesity Weight ‘Unhealthy adj4 weight’ ‘Healthy adj4 weight’ Pain management /Perception Attention, Memory Physical activity Physical inactivity Public health Restoration /Self- Care, Confidence, Efficacy, Esteem, Social isolation Sport Stress /Smoking Cessation, Tobacco use Water sports /Wellbeing Life satisfaction, Mood, Social cohesion, Social capital, Winter deaths

After removing duplicates, 536 potential studies were identified using this search protocol. Before the abstract review, two researchers (A1, A3) randomly chose 20 titles and independently reviewed abstracts based on the inclusion/exclusion criteria. There was 100% agreement on which titles to include and which to exclude. One researcher (A1) conducted abstract reviews; 83 were retained. Five studies were randomly chosen by a second researcher (A3) and reviewed for inclusion/exclusion; again there was complete agreement.

Charting variables for full text review were consistent with recommendations (Arksey and O’Malley 2005). They included: authors/date/journal, location and type (e.g. intervention, natural experiment), theoretical framework, project type (e.g. vacant lot greening, cycleway installation), sample/methods, health outcomes, main findings, and recommendations.

## Results

### Included studies

**Figure 1.**
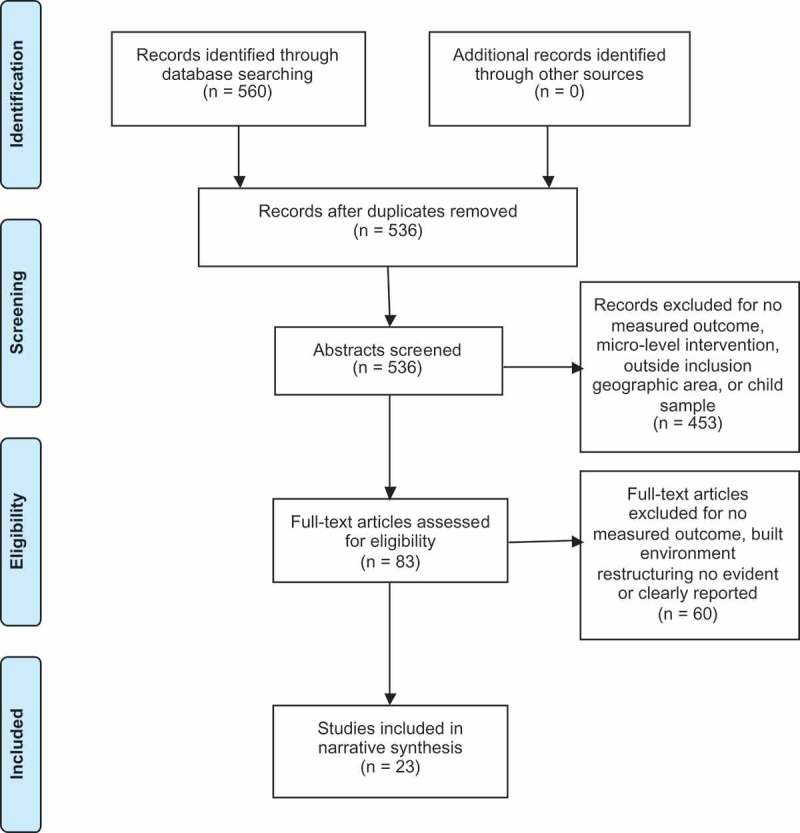
Flow diagram of extracted studies.

Of the 83 studies identified from the abstract review for full text review, 57 were excluded using the stated criteria and three because full text could not be obtained. The included studies (*N* = 23, Figure 1) represented 19 independent built environment initiatives. Of these 19, three were quasi-experimental studies, 15 natural experiments, and one assessed the impact of built environment zoning (i.e. planning) targeted to improve health behavior using nationwide data (Chriqui *et al*. 2016). Two of these presented findings from multi-city natural experiments (Ward Thompson *et al*. 2012, Goodman *et al*. 2013). Based on U.K. Medical Research Council guidance (Craig *et al*. 2012), natural experiments were classified as initiatives or interventions were there was no random assignment or experimental manipulation either did not occur or was not feasible under the circumstances. Quasi-experimental studies were those where there was some experimental manipulation by the researcher when investigating the impact of built environment changes; none were randomised control trials.

Studies were conducted in the USA (9), UK (5), Australia (3), Netherlands (1), and France (1). They were and reported in 17 journals, most of which were public health focused, although four were on urban design. Although the search timeframe was 17 years, the majority of studies (83%) were published in the last 5 years.

A summary of charted data for each study is provided in supplementary file 1. The narrative findings are presented by built environment project type, building development using New Urbanism design philosophy, health behaviours and outcomes affected, and methodologies used to assess the built environment. These findings are then integrated into the COM-B and TDF in the final sub-section of the results.

### Built environment restructuring by type

***World Health Organization Definitions of Physical Activity (2010***; ***2015***)

- ***Physical activity*** (PA) refers to movement classified in intensity based on ‘a ratio of working metabolic rate to resting metabolic rate’ (2015, p. 71).
- ***Moderate physical activity*** requires motion between 3–6 times the intensity of an individual’s resting metabolic rate, or a 5–6 self-rating on a 0–10 scale of effort.
- ***Vigorous physical activity*** typically refers to effort more than 6 times that resting rate, a self-rated effort of 7–8.
- ***Recommended guidelines*** for healthy adults (18–64) are 150 minutes each week of *moderate-to-vigorous physical activity* (MVPA) accumulated from sessions of at least 10 minutes; 300 minutes is considered necessary for increased health benefits.

#### Active travel

Of the included studies, 61% reported environment restructuring with the aim to improve active travel and/or physical activity (PA). Six studies assessed transport infrastructure change on active travel and other PA; projects included a bus network and traffic-free walking/cycling route (Panter *et al*. 2016), a cycle lane/sidewalk/light rail project (Miller *et al*. 2015, Brown *et al*. 2016), cycle/sidewalk/pedestrian safety/aesthetics project with promotional programmes and signage specifying shortest/pleasant routes (Buscail *et al*. 2016), a multi-city initiative to improve urban greening/parking provision/pedestrian safety (Ward Thompson *et al*. 2012), and a traffic calming scheme (Morrison *et al*. 2004). There were clear links between usage of the new provisions and more active commuting (Miller *et al*. 2015, Panter *et al*. 2016), increased time spent in commute-related PA (Miller *et al*. 2015, Panter *et al*. 2016), increased walking (Morrison *et al*. 2004), and increased moderate-to-vigorous physical activity (MVPA) (Buscail *et al*. 2016). Benefits were best for those living closest to the new provision (Brown *et al*. 2016, Panter *et al*. 2016) or who were previously least active (Panter *et al*. 2016). Other outcomes affected included improved perceived safety and fewer unhealthy days by the elderly (Ward Thompson *et al*. 2012) and better quality of life (Morrison *et al*. 2004, Ward Thompson *et al*. 2012). However, barriers such as nuisances (e.g. groups of youths, dog fouling) increased; and decreased parking availability near the home was important to elderly PA levels (Ward Thompson *et al*. 2012). In the light rail extension project, some residents stopped using public transportation to result in decreased PA (Miller *et al*. 2015). The authors did not investigate why this occurred, missing an opportunity to explore potential barriers to public transportation usage. Finally, efforts to educate the public about PA opportunities did not translate into greater awareness of them (Buscail *et al*. 2016), suggesting message content and type of information campaign are also necessary considerations to effect health behavior change.

One nationwide US study provided comprehensive information on the impact of urban planning, specifically zoning code reforms, on active commuting using American Community Survey data (Chriqui *et al*. 2016). Zoning code reform to improve sidewalks, cycle-pedestrian connectivity/infrastructure, street connectivity, mixed-used development, and walkability were investigated for their impact on walking, cycling, public transportation use, or any active commuting. The most common zoning reforms were sidewalks/walkability/pedestrian infrastructure (> 70%), mixed-use development (58%) and shared cycle-pedestrian trails (57%). Overall, the rate of active commuting in nearly 4000 municipal jurisdictions across 48 US state was low, with only 6.25% engaging in any active travel. Residents in areas implementing pedestrian/transit-oriented reforms used public transit more; and in jurisdictions implementing eight or more reforms, walking, cycling and active travel levels were highest. Both walking and cycling to work were higher in areas with cycle parking, bike/pedestrian paths, walkability initiatives, and mixed-use development. This study was included because it illustrated the role urban planning plays in creating *opportunity* for behavior change in lived built environments based on its comprehensive review of behavioral differences resulting from zoning reform from approximately 4,000 US municipal jurisdictions covering 73% of the US population.

Three studies targeted cycling. These improved active transport and recreation-related PA in both cyclists and pedestrians (Goodman *et al*. 2013, Crane *et al*. 2016), increased numbers of new cyclists (Crane *et al*. 2016), and decreased car commuting (Goodman *et al*. 2013). Workplace initiatives to promote cycling collectively explained 33% of the variation between locations in cycling prevalence (Goodman *et al*. 2013). These workplace initiatives included cycle parking, travel planning, cycling training, and building ‘cycling culture’, all of which could be considered facilitators of health behavior in behavioral science (Thaler and Sunstein 2008). Seeing others cycle influenced activity (Crane *et al*. 2016) and paths improved perceived social connectedness and area aesthetics, suggesting *social influences* and *beliefs/attitudes* within TDF (Cane *et al*. 2012) were affected.

However, not all impact was positive. Barriers included feeling unable or ‘too old’ to cycle and cyclist-pedestrian conflict occurred due to insufficient ‘rules of the road’ information leading to safety concerns and perceived rudeness by cyclists towards non-cyclists (Crane *et al*. 2016). Additionally, Dill *et al*. (2014) reported new cycle boulevards had no impact on MVPA or minutes spent walking, and actually decreased bike trips. They noted positive attitudes towards the activity were important, reinforcing the potential for *beliefs* and *attitudes* to influence health behavior (McEachan *et al*. 2011).

#### Urban greenways

Urban greenways are ‘physical connectors between areas with green cover’ (Sharma 2015, p. 26), often to town centres or areas of mixed-land use, designed to improve both recreational and commuting PA. Although many initiatives have been reported, we found only four studies with measurable outcomes as defined here. Greenways resulted in higher PA in intervention locations compared to control streets (Fitzhugh *et al*. 2010, Gustat *et al*. 2012). Using health economics modelling, two studies explored the potential impact of new urban greenways on future health based on current resident PA (Dallat *et al*. 2014, Longo *et al*. 2015). Initial indications were 35% of males and 53% of females were not meeting MVPA guidelines prior to the project (Dallat *et al*. 2014). Perceived walkability also predicted behavior (Longo *et al*. 2015); residents who perceived ‘good’ availability of shops and facilities walked 37 minutes more per week. The authors estimated improved walkability combined with information programmes targeted at resident perceptions would increase MVPA in inactive residents by 39 minutes per week and potentially reduce mortality by 8%. Both study authors suggested these projects can be cost-effective in increasing PA (Fitzhugh *et al*. 2010) and improving quality-adjusted life years through reduced disease incidence (Dallat *et al*. 2014).

#### Urban green space

We only identified four studies of urban green space (UGS) projects that included any measurable outcomes of interest. An outdoor gym installation combined with behavior change facilitators such as marketing, instruction sessions and instructional guides attracted new elderly users, increased their confidence, and users indicated intentions for future use and recommendations to friends (Scott *et al*. 2014). In socio-economically disadvantaged areas, creating small parks on single plots of land had a positive effect. Self-reported (Branas *et al*. 2011) and observed PA/MVPA (Cohen *et al*. 2014) increased, perceived safety improved (Cohen *et al*. 2014), stress and crime/incivilities declined (Branas *et al*. 2011). Yet, other comprehensive restructuring initiatives within low socio-economic areas including UGS refurbishments, new parks, and improved neighbourhood ‘green character’ showed little impact on PA (Droomers *et al*. 2016). These authors noted substantial variation in initiatives meant combined analysis could have obscured the impact of specific interventions. They also speculated a lack of change in PA levels could have been the result of residents *moving* their PA to an improved local area, replacing PA in another location.

### New Urbanism

The idea that built environments where people could live, work, and play support public health is a central tenet of New Urbanism (Day 2003). Features of these locations included mixed land usage, good walkability/active travel infrastructure, appropriate residential density, and parks/recreation space (Center for Active Design 2010). We included five studies describing three distinct New Urbanist locations that assessed measurable behavior change outcomes as specified by our criteria.

Across New Urbanist locations, residents engaged in more PA (Rodriguez *et al*. 2006, Calise *et al*. 2013, Christian *et al*. 2013, Zhu *et al*. 2013, Hooper *et al*. 2014) and more MVPA (Rodriguez *et al*. 2006, Zhu *et al*. 2013). Changes were most profound in those previously inactive or moving from less-walkable communities (Calise *et al*. 2013, Zhu *et al*. 2013). PA occurred *within the neighbourhood* more, suggesting design influenced where PA occurs (Rodriguez *et al*. 2006), as well as removing a barrier to PA (i.e. the need to travel) through better physical opportunity (Michie *et al*. 2011). Residents also reported better health after the move (Zhu *et al*. 2013), reduced social isolation, and reduced car journeys (Rodriguez *et al*. 2006, Zhu *et al*. 2013). New Urbanist design features varied in their impact (Hooper *et al*. 2014), with a neighbourhood centre complimented with higher-density housing increasing any walking and ≥ 60 minutes a week active transport; while better implementation of movement networks and land layout guidelines resulted in more recreational walking. Yet, across New Urbanist settings, the evidence supported their positive impact on PA, as well as the potential to increase social interaction and community cohesion, both important aspects of healthy cities (Swinburn *et al*. 1999, Barton and Grant 2006, 2013). Nonetheless, it is also important to explore the level of design implementation and the specific features associated with intended behavior change.

### Health behaviors and other outcomes

Despite exhaustive searches for a range of other health outcomes and behaviors, all studies focused on PA as the primary or sole outcome; and the measures used to operationalize it varied. Fourteen studies relied on self-reported PA using established questionnaires (e.g. Neighbourhood Physical Activity Questionnaire or Recent Physical Activity Questionnaire). Some determined if this was MVPA (Rodriguez *et al*. 2006, Calise *et al*. 2013, Longo *et al*. 2015, Panter *et al*. 2016) or if recommended weekly PA guidelines were met (Rodriguez *et al*. 2006, Dallat *et al*. 2014). Only four measured PA with accelerometers or GPS (Ward Thompson *et al*. 2012, Dill *et al*. 2014, Miller *et al*. 2015, Brown *et al*. 2016) and four observed PA in the study area (Morrison *et al*. 2004, Fitzhugh *et al*. 2010, Gustat *et al*. 2012, Cohen *et al*. 2014).

Other public health outcomes such as psychological and social health may also be affected by the built environment (Schultz *et al*. 2016). Several studies included subjective measures of stress, quality of life (Ward Thompson *et al*. 2012), general health (Branas *et al*. 2011, Ward Thompson *et al*. 2012, Droomers *et al*. 2016) and health-related quality of life (Morrison *et al*. 2004, Ward Thompson *et al*. 2012, Longo *et al*. 2015). Wider determinants of public health (PHE 2016) were affected by built environments and those reported here included social isolation and community cohesion (Zhu *et al*. 2013).

### Assessing built environments

The inclusion criteria required studies to include both built environment restructuring *and* measured outcomes/behaviors related to public health or behavior change. This requirement resulted in only a small number of studies being included. Of the 23 included, even fewer (5) included any assessment of the environment. Two studies implemented geographic information systems (GIS) technology to create indices of policy compliance with regional planning guidelines (Christian *et al*. 2013) and walkability (Hooper *et al*. 2014). Two others used walkability indices, one based on a formula combining land use mix, residential density, sidewalk density, and retail floor area (Longo *et al*. 2015) and the other on the Walkscore® method (Zhu *et al*. 2013), which is similar but uses proprietary software to calculate walkability.

### Integration with COM-B and TDF

In this section, the summary narrative findings are integrated with the components of the COM-B and domains of the TDF, in order to identify where behavioral science can potentially strengthen the design and evaluation of future built environment restructuring projects. Each included study was mapped onto the TDF domains within each relevant source of behavior change from COM-B (see Table 3). All studies provided *physical opportunity* (COM-B behavior source) via environmental context/resources (TDF domain). This was to be expected given that change to the physical environment was necessary in order to meet inclusion criterion for the scoping review. In the COM-B model (Michie *et al*. 2011), there are other sources of behavior most relevant to built environment restructuring. Seven studies included outcomes relevant to *social opportunity*, all which were related to social influence (TDF domain). For example, a New Urbanist community provided social opportunity through increased resident social interaction (Zhu *et al*. 2013) and another study reported initiatives to build a ‘cycling culture’ to encourage cycling to work (Goodman *et al*. 2013). *Automatic motivation* was evident in five studies, primarily through environment restructuring projects’ impact on perceived safety and stress (TDF domain: *emotion*) but also via reinforcement with *incentives* (Goodman *et al*. 2013). Other COM-B behavior sources were found in seven studies. Scott *et al*. (2014) reported elderly outdoor gym participants felt their skills (a TDF domain) for engaging in physical activity (COM-B *physical capability*) were improved by instructor-led sessions and information leaflets on how to use the facilities improved their knowledge (COM-B *psychological capability*). These influenced *reflective motivation* through increased confidence (TDF domain: beliefs) and future plans to use the gym (TDF domain: intention). Future intention was included in a study after the introduction of new bicycle infrastructure (Crane *et al*. 2016) and attitude towards walking/cycling (TDF domain: beliefs) predicted cycle path usage in another (Dill *et al*. 2014). In summary, although not explicitly integrated into the studies included in this review, it appears behavioral science techniques have been used.

**Table 3.** Integration of individual studies with TDF domains by COM-B behaviour source.

	COM-B model behaviour sources^a^ associated with environmental restructuring			Other COM-B model behaviour sources^a^		
Author by built environment restructuring type	Automatic motivation^b^	Social opportunity^b^	Physical opportunity^b^	Psychological capability^b^	Physical capability^b^	Reflective motivation^b^
**Active Travel**						
Brown *et al*. 2016			Environmental Context/Resources			
Buscail *et al*. 2016			Environmental Context/Resources	Knowledge		
Chriqui *et al*. 2016			Environmental Context/Resources			
Crane *et al*. 2016		Social Influences	Environmental Context/Resources			Intentions
Dill *et al*. 2014			Environmental Context/Resources			Beliefs
Goodman *et al*. 2013	Reinforcement	Social Influences	Environmental Context/Resources	Behaviour Regulation; Knowledge	Skills	Goals
Miller *et al*. 2015			Environmental Context/Resources			
Morrison *et al*. 2004		Social Influences	Environmental Context/Resources			
Panter *et al*. 2016			Environmental Context/Resources			
Ward Thompson *et al*. 2012		Social Influences	Environmental Context/Resources			Beliefs
**Urban Greenways**						
Dallat *et al*. 2014			Environmental Context/Resources			
Fitzhugh *et al*. 2010			Environmental Context/Resources			
Gustat *et al*. 2012			Environmental Context/Resources			
Longo *et al*. 2015	Emotion		Environmental Context/Resources			Beliefs
**Urban Green Space**						
Branas *et al*. 2011	Emotion		Environmental Context/Resources			
Cohen *et al*. 2014	Emotion	Social Influences	Environmental Context/Resources			
Droomers *et al*. 2016			Environmental Context/Resources			
Scott *et al*. 2014		Social Influences	Environmental Context/Resources	Knowledge	Skills	Beliefs; Intentions
**New Urbanism**						
Calise *et al*. 2013			Environmental Context/Resources			
Christian *et al*. 2013	Emotion		Environmental Context/Resources			
Hooper *et al*. 2014			Environmental Context/Resources			
Rodriguez *et al*. 2006			Environmental Context/Resources			
Zhu *et al*. 2013		Social Influences	Environmental Context/Resources			

^a^Definitions of each COM-B behaviour source were obtained from http://www.behaviourchangewheel.com retrieved 21 July 2017.

^b^ Each behaviour change source can include a range of TDF domains (Cane *et al*. 2012). For example, automatic motivation as a behaviour change source in the COM-B model includes social/professional identity, optimism, reinforcement, and emotion TDF domains. Each TDF domain may then include up to 11 different concepts, therefore only the higher order TDF domains were mapped here. Where other behaviour change sources of the COM-B model were observed, they were also included.

## Discussion

The aim of this scoping review was to determine whether two common approaches for public health improvement, built environment restructuring and behavioral science, could or should be integrated. Our findings indicated built environment researchers were already using a number of behavioral science outcomes consistent with the COM-B and TDF, but not necessarily intentionally. The obvious reason for this was the projects reviewed here were developed prior to the initial publication of either COM-B or TDF. However, this cannot explain the lack of reports of the theories underpinning these built environment interventions. With the exception of one study based on the Theory of Planned Behavior (Crane *et al*. 2016), there was no evidence that behavioral science was intentionally integrated into project design; and only four other studies mentioned any theoretical framework. Three reports (Rodriguez *et al*. 2006, Ward Thompson *et al*. 2012, Zhu *et al*. 2013) were based on socio-ecological theory (McLeroy *et al*. 1988) and one on Broken Windows Theory (Branas *et al*. 2011). While we acknowledge reporting conventions vary across disciplines and, therefore, this information may not be required, we reiterate the need for theory development recently raised by others (Hassen and Kaufman 2016); and go further to suggest that the theory underpinning design should be explicitly communicated in all published accounts.

We would also recommend that built environment restructuring projects have health outcomes integrated from the initial design stage and based on clearly specified theoretical framework(s). A clear challenge in this respect is the need to include theories that bridge individual behavior through to macro-environment influences (Tate *et al*. 2016). The Health Map (Barton and Grant 2006, 2013) provides a useful framework to this aim but does not provide theoretical linkages. Based on the evidence presented in this review, we propose behavioral science frameworks such as the COM-B (Michie *et al*. 2011, 2014) and TDF (Cane *et al*. 2012) are useful at the individual-level; but that future cross-disciplinary collaboration is needed to synthesize theoretical approaches targeting different levels of influence into a multi-level, integrated theoretical model.

From a behavioral science perspective, built environment restructuring provides the physical opportunity for behavior change (Michie *et al*. 2011) through environmental context and resources (Cane *et al*. 2012). This review clearly indicated these physical opportunities typically translated into improved physical activity; but due to variability in methods used to measure physical activity, comparison across interventions was not possible. Reliance on self-report data is also particularly problematic in the behavioral science context, given self-reports often over-estimate PA (Troiano *et al*. 2014).

Being ‘healthy’ is based on multiple factors and built environment research should reflect a wider breadth of health outcomes and behaviors. In our review, we found evidence for the positive impact on stress, general/health-related quality of life and social isolation. Subjective environmental perceptions and attitudes can influence the use of urban settings, particularly green resources (Flowers *et al*. 2016) and should be reported in conjunction with physical activity. Other sources of behavior such as motivation, social opportunity, and beliefs about physical and psychological capabilities should also be acknowledged as important drivers to the health behavior these projects might intend to target.

Conversely, little research has focused on potential negative consequences resulting from built environment restructuring. Some evidence was presented indicating conflicts between user groups occur (Crane *et al*. 2016) and nuisances (e.g. dog fouling, groups of youths gathering) can arise (Ward Thompson *et al*. 2012). These findings reiterate the range of potential negative health and wellbeing outcomes associated with urban design factors such as noise, poor design quality, crowding and density, which have been summarized by other authors (Cooper 2014). Further research exploring these negative consequences has also been suggested by the WHO (2017); and, despite our focus in this review on measurable outcomes, we also argue for a balance between quantitative studies gathering self-report and observational behavior data with qualitative research exploring barriers and facilitators of healthy behavior in built environments.

In regards to the assessment of built environment characteristics, very few studies did so and there were potential limitations to the methods implemented. Walkscore® (Zhu *et al*. 2013), as a measure of walkability, only measures distance; however, multiple factors impact people’s propensity to walk. Other methods may also be potentially problematic. For example, a measure of land use mix may show as ‘mixed use’ based on a residential area with a drive-through restaurant and commercial warehousing but it will not necessarily support walking. The lack of consistency in measures of the built environment and the inability of them to be nuanced enough to be helpful is a criticism that has been made many times (Townshend and Lake 2009). There are also substantive problems with regard to ‘assessing’ the quality of green space within the urban context. GreenSpace Scotland (2008) defines quality greenspace as greenspace which is ‘fit for purpose’ – meaning it is in the right place, readily accessible, safe, inclusive, welcoming, well maintained, well managed and performing an identified function. Combining these complex variables would enhance usage. Therefore, it is essential to include measures of quality and quantity whilst attempting to avoid unwieldy research designs.

Finally, we suggest an approach to incorporate many of these recommendations. Built environment intervention project teams should agree the relevant theoretical frameworks from their respective disciplines in the initial planning stage. The manner by which these frameworks inform study design should then be explicitly summarised in a published study protocol. Study protocols are common in the public health, with examples of some that bridge with urban design (e.g. Chapman *et al*. 2014) and include how theory underpinned design (Razani *et al*. 2016). Published protocols should then be referred to in all subsequent published accounts, thus avoiding the need to fully summarise this information further. During the design stage, a variety of outcomes should be defined covering the breadth of both built environment and health evaluation needs. This could include assessing built environment characteristics and public perceptions of these settings, measuring health outcomes through objective means such as GPS tracking of physical activity, and capturing subjective health and wellbeing outcomes using *both* internationally-recognised measures (e.g. health-related quality of life, social isolation) *and* qualitative exploration of the users lived experience. The intention of our suggested approach is not to introduce unnecessary or unwieldy theoretical complexity to projects. Rather, our intention is to develop the ability to compare the effectiveness of interventions across settings or types of built environment interventions, as well as with other health behaviour change initiatives targeting the same health outcomes. The recommended approach also facilitates linkages between published accounts, each focused on a subset of discipline-specific results, to provide a full picture of both the positive and negative consequences of built environment interventions.

### Study strengths and limitations

The primary strength of this scoping review was its attempt to integrate two disparate, yet common approaches to improving public health and health behaviors. Recent reports reiterate the potential for built environment and behavioral science approaches to facilitate our understanding of the varied, multi-level influences on health (Hollands *et al*. 2013, Arnott *et al*. 2014, Roberts *et al*. 2016); and our integration of the summary findings with two established behavioral science frameworks, the COM-B and TDF, further support the potential for synergy between them. It is important to acknowledge that scoping reviews are not without their limitations. As an evidence review method, the aim is to summarize evidence in a time-limited context. This required a decision to focus only on peer-reviewed studies, which meant potentially relevant sources such as government reports were absent. This is potentially problematic in two ways. First, design and evaluation phases of these types of interventions are not necessarily implemented by the same stakeholders. This may mean those who conduct these evaluations are inadvertently unaware of the theories underpinning original intervention design. The sole use of peer-reviewed published accounts and exclusion of grey literature such as government agency reports also means that potentially valuable lessons from implemented interventions were not included. Yet, we believe these concerns reinforce our recommendation that published protocols for interventions should become best practice. Even if later evaluations were only reported in the grey literature, the published protocols would facilitate compiling the relevant evidence for a specific project as the grey literature can cite the protocol. Overall, we believe the search parameters implemented in 12 academic databases across three disciplines minimised this limitation and provided the most thorough results possible.

Another limitation of this review was the decision to focus on studies only reporting measurable health outcomes and behaviors. In doing so, it was likely that informative studies reporting only subjective outcomes relevant to behavior change were excluded. Future reviews could focus on a single or limited number of similar built environment restructuring interventions and synthesise the range of behavioral science-relevant outcomes across published reports.

As with other health-related outcomes, it is also possible that detailed accounts of environment evaluations were presented in other published studies that did not meet the inclusion criterion for this scoping review. For example, studies would have been excluded because they focused on resident perceptions of the lived environment (but included no behavior) or were solely focused on evaluation of the design features. Therefore, we believe it is important for authors to either provide some account of this information in all studies or at the very least refer readers to other published studies in order to understand the full impact of these design initiatives.

Additionally, our initial search strategy was somewhat constrained by the requirements of the funder in respect of their wider remit to inform its future research priorities. As a result, the search was focused on studies from locations similar to the UK but did include a diverse number of countries. The funder, however, did not contribute to the current scoping review and encouraged us to disseminate any more specific, independent findings that resulted from the wider review.

## Conclusion

The capacity for built environment restructuring to positively impact public health was clear in the studies reported in this scoping review; however the pathways for this impact remain unclear. In part, this is because existing evidence is too focused on physical health and there is a need to look at these pathways linking the individual, the environment, and their health, including mental health, more holistically. Nonetheless, good evidence is emerging that built environment interventions can facilitate improved public health, that these initiatives may be strengthened by integration with behavioral science.
