# Supplementary material for: Restructuring the built environment to change adult health behaviors: a scoping review integrated with behavior change frameworks
Source: Cities Health. 2019 Feb 20;2(2):198–211. doi: 10.1080/23748834.2019.1574954 (PMC6777541; doi:10.1080/23748834.2019.1574954)
Supplement: Supplemental Material [file RCAH_A_1574954_SM4464.pdf]

Author	Date	Journal	Study Location	Study Type	Theoretical Framework	Project Type	Study Aim(s)	Sample	Method	Measured Behaviour Change Outcome(s)	Other Public Health Related Outcomes	Main Findings	Gaps/recommendations
Branas et al.	2011	Am Jn Epidem	Philladelphia, USA	Natural experiment	Broken windows theory; Incivilities theory	Greening vacant lots	Assess programme for long term health effects	State Household Health Survey. Vacant & matched control lots.	Survey; Difference-in-difference analysis	Low exercise = less than 2 30-min. physical activity (PA) sessions/week	Diagnosed with high cholesterol/blood pressure by doctor; Stress (high - 10) in past year.	Greening reduced stress and increased exercise. The impact on other health was mixed across locations.	The underlying reasons vacant lot greening impacts health is not clear. Recommend mixed methods including ethnographic methods.
Brown et al.	2016	J PhyAct Health	Salt Lake City, USA	Natural experiment	None listed	Light rail extension; Better/more complete cycle paths; Wider sidewalks; Improved aesthetics to sidewalks	Assess change in PA and commuting behaviour	Near (<800 m) and far (>800 m) to street renovation; English or Spanish; Able to walk a few blocks; 1 year pre-post (Pre *N* 910; Post *N* = 536)	Survey; GPS; Accelerometer	Transit & non-transit bike trip (time and distance)	--	Residents living near to light rail increased active transportation from 20 to 25%. Those living far did not. Both groups reported increased walking. Increase in transit use was mostly by ummarried, nonwhite residents with no car. Cyclists more likely to be white males with no car.	Some users lived far from the rail extension yet made the effort to access new transit options. Research should explore what characteristics encouraged this to identify facilitators of this behaviour.
Buscail et al	2016	BMC Pub Health	Paris, France	Quasi-experimental study	None mentioned	Pedestrian paths; Bike path; Widened sidewalks; Speed bumps	Assess intervention impact on PA	Stratified random cluster sampling among dwellings; 2 years pre-post (Pre *N* = 199; post *N* = 217)	Survey	Meet World Health Organization recommended weekly MVPA (yes/no): overall, leisure, work	Barriers & levers to PA in location	The number of residents reaching sufficient PA levels increased by 15%. Walking increased due to increased walking by women for leisure. Information campaigns combined with environment interventions successfully in raised some awareness of project but there were still opportunities to improve communication of PA opportunities in the area.	--
Calise et al 2013	2013	Prev Chronic Disease	Austin, USA	Natural Experiment	None listed	New Urbanist Community	Change in PA post-move; Examined neighborhood preferences	1 adult per occupied household, free from mobility issues (*N* = 267). All data collected post-move. Pre-move data was retrospective self-report	Survey	Total minutes PA/week; Total minutes transport related PA (cycle, walk)/week; Total minutes recreational cycle & walk/week; Total recreational PA (total walk/bike + other moderate-vigorous PA MVPA)	Neighbourhood ratings: affordability/value, closeness to open space/job/school/public transport/shops/services/restaurants/recreational facilities/health care/entertainment choices, ease of walking, sense of community, school quality, motorway access	Most post-move PA was recreational. Recreational walking *in the neighbourhood* increased and outsidethe neighbourhood decreased post-move. Respondents reporting high levels of activity pre-move were still the most active. Significant increasedpost-move total and recreational PA were in pre-move low and moderate activity groups.	There was no exploration of reasons residents choose this community. Suggest future studies should use prospective design and explore individual differences.
Chriqui et al.	2016	Front Pub Hlth	National, USA	Planning analysis	None listed	Pedestrian-oriented planning codes	Investigate links between active travel and zoning (i.e. planning) codes	3914 jurisdictions in 472 counties & 3 cities, 48 states & DC; American Community Survey respondents	Policy review; Survey	Active travel (walking, bike, public transport) to work in last week; Any form of active transport to work	Walkability scale: 4-way intersections, intersection density, # intersections/land area, housing unit density, population density	Overall, low reported levels of active travel. Zoning (i.e. planning) measures increased bike use. Mixed-use zoning increased walking.	There is limited literature on the impact of zoning/planning and active health outcomes but the findings suggest it is an important means to effecting change.
Christian et al.	2013	AM J Pub Hlth	Australia	Natural Experiment	None listed	"Livable" Neighboorhoods	Determine impact of planning code implementation on walking	English speaking; planned to relocate to either a livable, hybrid, or conventional neighbourhood; recruited from water authority (as part of move process). Pre move, 12 months, 36 months (N = 1047)	Survey	Change in total weekly minutes of recreational, transport, & total walking	Neighborhood perceptions: access to mixed-use services, street connectivity, traffic safety, crime safety, neighbourhood aesthetics. Neighbourhood selection reasons: pedestrian/cycle friendly streets, access to services/jobs/school, close to parks/recreational facilities, safe/diverse community	People chose livable or hybrid neighborhoods because of walkability characteristic. Transportation walking did not increase over time or differ between neighbourhoods. Recreational and total walking was higher in livable and hybrid neighbourhood residents. Walking in control neighbourhoods was high at baseline and did not increase; thus suggesting moving to liveable or hybrid neighbourhoods had the intended effect. Residents of the livable neighbourhoods also viewed it more positively than control residents views of their neighbourhood.	Future studies should look at amount of planning code implemented and dose of exposure.

Cohen et al.	2014	Am J Pub Hlth	Los Angeles, USA	Natural Experiment	None listed	New pocket parks	Assess impact on PA; Compare PA levels between pocket and existing parks	Low SES, ethnic minority; Lived w/in 0.5 miles; Matched pocket park to control parks; Pre (*N* = 392); Post (*N* = 432)	Observations; Interviews; Survey (random sample)	Park use; Observed MVPA; Estimated metabolic output of PA (METS); Self-report park visits; Self-reported PA in past week	Cost effectiveness of park construction amortized over 30 years; $ per METS hour per yr.	Pocket parks users were often sedentary (e.g. observing children at play) but people walked to the park (average 0.25 miles). Pocket parks encouraged social interaction and were good value for investment.	Programmes and events should be used to raise awareness of the new parks. Exercise classes which target MVPA could be held outdoors in these smaller locations.
Crane et al.	2016	Jn Trnsprt Hlth	Sydney, Australia	Natural Experiment	Theory of Planned Behavior	Installation of 2.4 km cycleway	Impact on cycling; Changes in perception, attitude	Interviews Residents (N = 12) & Retailers (N = 13) 1 year pre-post; Cycleway Survey (*N* = 783)	Survey; Intercept interviews	Cycleway use = purpose, time, origin, destination; Intention to use cycleway; Facilitators & barriers to use	Perceptions of: impact to health/leisure/commute behaviour/overall QofL/community connection	People changed route to use new cycleway, which was primarily used for commuting. 18% of survey respondents were new users. Seeing people cycling influenced others to increase activity. Rresidents also perceived improved aesthetics and connection with neighbors. Conflict between cyclists & pedestrians occured because road rules were not clearly communicated. Barriers to cycle path use were no bicycle, safety concerns, lack of awareness, and no cycling experience.	There were perceptions of the cycleway as a commuter route even though 1/3 of users were non-commuters. Attitudes towards cycling can also be barriers to use and should be explored.
Dallat et al.	2014	Eur Jn Pub Hlth	Belfast, UK	Natural Experiment	None listed	Community Greenway	Estimate potential health benefits & cost effectiveness using PREVENT model of annual costs for Type 2 diabetes, cardiovascular disease, colon/breast cancer	Household survey (*N* =1209); 63% response rate	Survey; Modeling	PA (mins. per week); Proportion meeting 150 MVPA recommendation	Gains in life expectancy (LE); Disability adjusted LE (DALE); Years lived with disability (YDL): Scenario modeling: A 2% inactive become active; B 5%, C 10%	All scenarios are cost-effective and reduce incidence and mortality, with scenario C the most effective. Incremental cost effectiveness ratios ranged £4469 to £18411 DALY. Modelled increased walking due to the project was predicted to have the most impact on preventing diabetes and ischemic heart conditions.	Intrapersonal and environmental factors must be explored. This should include subgroup analyses, particular with regard to low SES, to reduce health inequalities.
Dill et al.	2014	Prev Med	Portland, USA	Natural Experiment		Bicycle boulevard installation	Change in physical activity, active transport usage; Control of demographics & attitudes	Family Activity Study; Able to cycle & have a bycycle (Pre *N* = 490; Post *N* = 353); 72% retention rate	Survey; GPS; Accelerometer: Difference-in-difference analysis	Minutes MVPA based on GPS & accelerometer data	Attitudes towards cycling, walking, driving	Bike boulevard Installation did not increase MVP. Attitudes towards cycling did predict cycling behaviour.	Time between completion of project and behaviour change should be considered. Boulevards varied in their design based on local needs and future studies should explore the effect of this variation.
Droomers et al.	2016	J Epidemiol Comm Hlth	Multi-site, Netherlands	Natural Experiment	None listed	Varied (e.g. small parks, vacant lot conversion to large park, landscaping, path installation)	Impact of project on PA & perceived health	Dutch National Health Survey (Intervention neighbourhood *N* = 1018)	Computer assisted personal or telephone interview	Active at least once a week; Frequency & mins. per day walking, cycling, sports in last week	Self-reported general health	There were mixed findings. Walking increased overall but did not differ between intervention and control areas. The highest activity increases were in low SES areas with no environmental changes. Cycling increased in intervention locations but no impact on perceived general health.	Green interventions tailed to specific locations were reported precluding an evaluation of intervention types generally.
Fitzhugh et al.	2010	Am J Prev Med	Knoxville, USA	Natural Experiment	None listed	Urban greenway/trail to improve connectivity with schools/amenities	Assess intervention impact on PA	Public activity observations over 2 hour period; conducted in intervention & control neighbourhood; 6 months prior to construction; follow up observations up to 2 years later		Counts of persons walking, cycling, other PA	--	Total PA increased post-intervention compared with control neighbourhoods.	The study design specifically excluded the use of social marketing or awareness campaigns. Study design also precluded determining whether users were new or how they differed demographically.
Goodman et al.	2013	Soc Sci & Med	Multi-site, England, UK	Natural Experiment	None listed	"Cycling demonstration towns" and "Cycling Cities & Towns"	Compared cycling to work in intervention towns with matched control towns; Change in walking, driving, public transportation use; Impact of deprivation	Commuters in 18 towns (*N* = 1.3 million)	National Census	Cycling to work: Proportion reporting cycling as main transport mode; Prevalence of walking to work; commute by car/van/motorcycle;Pp ublic transport to work	--	Cycling to work in intervention towns increased by 0.69 percent; walking to work increased by 1.71 percent compared to control towns. Increased cycling was observed more in low SES areas and local initiatives may increase that impact.	No randomization means limited to no causality conclusions; this is difficult to do but important to attempt in future studies.

Gustat et al.	2012	Prev Chronic Disease	New Orleans, USA	Quasi-experimental study	None listed	Installation of 8-foot wide walk paths to connect park with commercial corridor	Determine change in physical activity	Low SES, ethnic minority and control neighborhoods; Household stratified random sample; 2 years (pre - post) (Pre *N* = 449; Post *N* = 692)	Survey	Self-reported walking for leisure, transport, other PA; Observed number of persons sedentary, engaging in MPA or VPA	--	There were observed Increases in observed MPA and VPA in intervention area.	Decisions on where and how to provide opportunity for increased PA should include resident participation.
Hooper et al.	2014	Am J Hlth Prom	Australia	Natural experiment; Policy implementation	None listed	"Livable" Neighbourhood Design	Link between policy implementation & walking	Housing development residents (*N* = 594); 5 - 6 years post move	Survey	Any walking, >60 mins. for transport and rec walking (4 variables)	Policy implementation summed score across several areas	No area implemented all policy outcomes; on average only half were implemented. Each 10% increase in implementing movement network policies increased walking for transport by 2.48 (OR). Compliance with community design and lot layout elements increased active transport and recreational walking.	Need to ensure policy implementation is part of evaluation.
Longo et al.	2015	Soc Sci & Med	Belfast UK	Natural Experiment	None listed	Community Greenway	Determine neighbourhood improvements impact on walking; Determine monetary value of increased walking	Household survey; adults (*N* =1209; 63% response rate)	In-person Interview	Minutes walking in/outside of neighborhood per week; Mins MVPA per day	Health-related quality of life; Objective walkability: safety/walking paths/local shops; Perceived walkability	Residents perception of "good availability of shops" resulted in 36.48 more minutes walking each week. The authors estimate improving perceptions of inactive respondents could improve PA by 35%	Researchers need information on monetary health benefits. The WHO HEAT model underestimated total value of walking compared to implemented model. Future studies should focus on transitioning inactive residents to meeting recommended guidelines.
Miller et al.	2015	Health & Place	Salt Lake City, USA	Natural experiment	None listed	Light rail extension; better/more complete cycle paths; wider sidewalks; improved aesthetics to sidewalks	Explore if travel to new transit stations replaces other physical activity	Door to door canvassing; must be able to walk 2+ blocks; 1 year pre-post (N = 536)	Survey; GPS; Accelerometer	Light to moderate transit PA (min 5 mins. duration with min 1000 accelerometer counts per min); Moderate to Vigorous PA (MVPA); Total PA	Public transit use: never, continued (from before intervention), new, former	Public transit generates new PA, especially light rail compared to bus. Total PA increased for new users but decreased for former public transit users. Diversity and density of activities around transit shops should be considered.	Public health should be part of transit planning.
Morrison et al.	2004	J Epidemiol Comm Hlth	Glasgow, UK	Natural experiment	None listed	Traffic calming measure on on main road of deprived	Assess scheme impact	Random sample from households (*N* = 185)	Survey; Observation	Observed walking	Health-related quality of life	Observed pedestrian activity increased and reported physical health-related quality of life also improved.	It is important to consider the health and social impacts of built environment interventions in addition to evaluating original project delivery aims.
Panter et al.	2016	Am J Prev Med	Cambridge, UK	Natural Experiment	None listed	Busway Intervention	Explore change over time	Worked in city on busway; Lived w/in 30km (*N* = 469 at 1 year post-opening follow up)	Annual survey	Travel modes used in last week; Total weekly time walk/cycle for recreation/commute; Total time recreation MVPA; Total time overall PA	--	Closer exposure to busway increased cycle commuting time (mean = 87 mins. per week) but did not impact walking, total recreational, or overall PA.	Natural experiments are crucial to the evidence base between infrastructure changes, policymaking, and health.
Rodriguez et al.	2006	J Am Plan Assoc	Chapel Hill & Carrboro, USA	Natural Experiment	Socio-ecological Model	New Urbanist Community Design	Relationship between new urban neighborhood & PA	Head of households; Matched communities (*N* = 370)	Survey	Total MVPA; Meet recommended MVPA per week (yes/no); Inactivity	PA by location (home, neighborhood, outside neighborhood); Travel diary: time, destination, mode, distance,	There were no differences in MVPA between new urbanist or traditional neighbourhood residents. New urbanist residents engaged in more PA *within their neighbourhood*. Travel diaries suggested more walking and cycling in new urban neighborhoods, as well as reduced car use.	"Time budgets" for PA are an important factor to consider as people may not have more time to complete PA despite increased built environment provision. This may result in a substitute effect where PA in one context replaces another.

Scott et al.	2014	Hlth Prom Jn Aus	Australia	Quasi-experimental study	None listed	Outdoor gym	Evaluate intervention effectiveness; Explore impact of marketing and social support programmes	Session attendees; Mostly 50+; Sessions over 8 weeks (N = 66; 50% response rate)	Survey	Change in use; Confidence in using gym; Barriers & facilitators to use; Intended future use	--	The outdoor gym and instruction sessions attracted new users and increased confidence. Instructor sessions were an important facilitator and the guide to using the equipment could supported continued use. The intervention was more effective for older women than men. Advertising and 'walking past' while activities taking place raised awareness.	Equipment installation should be appropriate for older adults, with a mix of types (e.g. aerobic, balance, strength) and near to other PA infrastructure.
Ward Thompson et al.	2014	Br J Sports Med	UK excl. N. Ireland	Natural Experiment	Social-ecological Theory	Sustran Livable Neighborhoods DIY Streets (planters, parking provision changes, traffic calming)	Change in physical activity	Intervention & comparison street across 9 sites; 3-6 months post intervention (N = 61; est. 50% pop)	Survey; Accelerometer	PA intensity (counts/minute); Frequency of outdoor visits; Typical time spent outdoors for necessary walking/recreational walking/other outdoor activities; EQ-5D	Quality of life; Health-related quality of life; Neighbourhood perceptions; Change in social connections; Reported unhealthy days	Attractive and easy access open space influences elderly activity. The intervention group felt it was easier to walk on their street but did not report being more active, possibly due to declining health.	The potential for interventions to negatively impact activity (e.g. lack of car parking space) should be considered.
Zhu et al.	2014	Prev Med	Austin, USA	Natural Experiment	Social-ecological Theory	New Urbanist Community Design	Pre-post (retrospective) change in PA, social interaction, cohesion; variation by subgroup	Occupied housing units (N = 449); 36.3% response rate)	Survey	Days per week 30 mins. MPA; Cycling (mins./week); Total walking (mins./week); Walking in community (mins./week); Car travel (mins./week)	Social interaction frequency; Social cohesion; Walkability	The change in PA was for mostly people moving from low walkable areas to new area. Social interaction and cohesion increased.	Longitudinal studies with control groups should be implemented if possible.

PA = Physical activity; MVPA = Moderate-to-vigorous physical activity; MPA = Moderate physical activity; VPA = Vigourous physical activity
